# Effect of Respiratory Rehabilitation Before Open Cardiac Surgery on Respiratory Function: A Randomized Clinical Trial

**DOI:** 10.15171/jcvtr.2015.03

**Published:** 2015-03-29

**Authors:** Seyed Kazem Shakouri, Yaghoub Salekzamani, Ali Taghizadieh, Hamed Sabbagh-Jadid, Jamal Soleymani, Leyla Sahebi, Roya Sahebi

**Affiliations:** ^1^ Physical Medicine and Rehabilitation Research Center, Tabriz University of Medical Sciences, Tabriz, Iran; ^2^ Tuberculosis and Lung Disease Research Center, Tabriz University of Medical Sciences, Tabriz, Iran; ^3^ Tabriz Health Service Management Research Center, National public health management center (NPMC), Tabriz University of Medical Sciences, Tabriz, Iran

**Keywords:** Coronary Artery Bypass, Respiratory Rehabilitation, Arterial Blood Gas, Spirometry

## Abstract

***Introduction:*** Prevention of pulmonary complications after coronary artery bypass graft is attended as a very important issue. The aim of this study was to evaluate the role of pulmonary rehabilitation before surgery for reducing the risk of pulmonary complications after surgery.

***Methods:*** In a randomized clinical trial, 60 patients undergoing heart surgery were randomly divided into two groups A and B. Chest physiotherapy was performed before and after surgery on group A patients however it was done on group B’s, only after surgery. Effects of preoperative pulmonary rehabilitation were compared between two groups, using spirometry and arterial blood gas (ABG).

***Results:*** Thirty nine males (65%) and 21 females (35%) with mean age of 8.10 ± 9.56 were analyzed. The mean differences were statistically significant for predicted forced vital capacity (FVC) (CI 95%:1.3 to 8.7) and Predicted Peak Flow indices (PEF) (CI 95%: 1.9 to 9.4) of spirometry indicator, PCO2 index (of ABG parameter) (CI 95%: 1.4 to 8.9) and mean oxygen saturation (mean Spo2) (CI 95%: 0.6 to 1.7) of ABG index in two groups.

***Conclusion:*** The performance of pulmonary rehabilitation program before surgery is recommended, as it may result in the reduction of complications of heart surgery

## Introduction

Coronary artery disease (CAD) was introduced as the most prevalent cause of mortality all over the world in 2009.^1^ In order to cope the disease, coronary artery bypass grafting (CABG) was introduced as a novel advanced treatment in which, the patient undergoes general anesthesia. Usually the surgeon carries out sternotomy procedure and then the patient is attached to a cardio-respiratory bypass machine.^2^ There is a high incidence of pulmonary complications (PC) after such surgeries.^3^ The global average prevalence of PC following CABG surgery was estimated to be 2-4% and the most common complications were reported as: atelectasis (27-95%), pleural effusion (16.6-88%) and phrenic nerve paralysis (30-75%).^4^ Probability of PC following the CABG is not clearly determined in Iran, however the incidence of PC following the general surgery has been reported as 50%, approximately.^5,6^ The PC after cardiac surgery leads to heavy socio-economic burdens such as prolonged hospitalization, ICU admission, enormous costs of treatment, loss of the work days and even death, if the patient is not properly managed.^1^ Several conventional techniques have been proposed in the control of lung function postoperative, including the mucus suction, airway positive pressure exercise, physical therapy, to name a few.^5^ At present, various thoracic cavity physiotherapy techniques are used in order to increase the respiratory volumes, improve oxygenation and decrease respiratory complications after CABG^7^; however, despite the common use of respiratory exercises in patients after CABG in different countries, there is still insufficient scientific evidence for their efficacy.^8^ Due to high prevalence of coronary diseases, their life-threatening nature, the need for surgical intervention in the majority of such patients and also the probability of high incidence of PC in patients undergoing such surgeries, it is absolutely necessary to evaluate the effect of respiratory rehabilitation on prevention of complications after surgery. In addition, the lack of sufficient information about the effect of preoperative rehabilitation on the rapid recovery and management of complications emphasizes the importance as well as necessity of such a study. The aim of this study was to survey the effect of preoperative respiratory rehabilitation in patients undergoing open cardiac surgery.

## Materials and methods

### Design

In this simple randomized clinical trial was performed blinding on Outcome measurements (spirometry and 6 MWT) assessor and statistician. The patients were asked to take part in the study after they received full explanations about the aim of study, random allocation to study groups and reassurance about the absence of any side effects. The patients were randomly assigned to the intervention (group 1) and control (group 2) groups. Collection of subjects lasted approximately 12 months. The reliability of the study was evaluated and confirmed by a physiotherapist and an evaluator who had been fully informed of the study by the researcher. The equipment used for the physiotherapy and evaluation of the respiratory function held the required standards for physiotherapy and was used under the direct supervision of the principal investigator in the present study.

The patients in group A underwent physiotherapy 15 days before the surgical operation, with an emphasis on strengthening inspiratory muscles, and thoracic cavity physiotherapy was carried out based on the surgical ward routines. However, patients in group B received only postoperative physiotherapy based on surgical ward routines.

### Participants

**Figure 1:**
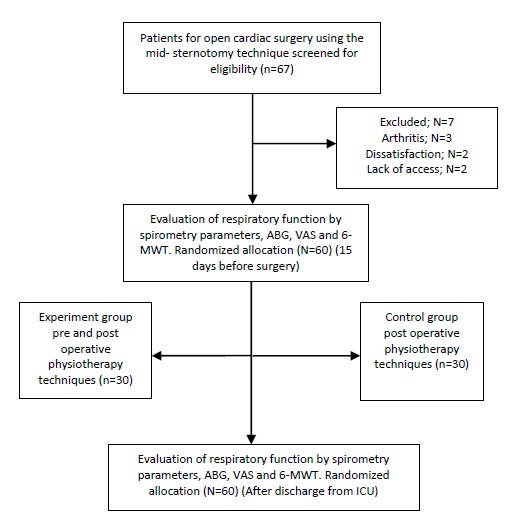
Design and flow of patients through the trial

Sixty seven patients were candidates for open cardiac surgery using the mid-sternotomy technique in Tabriz Cardiac Educational/Treatment Center and had similar conditions regarding the administration of medicines. A total of 60 CABG patients were included in the present randomized clinical trial in a period of 12 months among 2011-2012 (Figure 1). The exclusion criteria consisted of a history of any chronic respiratory condition, emergency surgery, cardiac insufficiency (EF<40%), valvular disorders, history of connective tissue conditions, chronic renal insufficiency, and a history of musculoskeletal disorders.

### Intervention

Preoperative physiotherapy techniques for group A consisted of the following:

1. Breathing exercises consisting of 10 deep breathing attempts, diaphragmatic breathing and pursing of the lips;
2. Instruction of flow-IS-based incentive spirometer (Respiflow™ FS) and effective coughing;
3. Instruction of neck and shoulder mobilization exercises with an emphasis on thoracic extension and rotation;
4. Instruction of muscular tension exercises;
5. Instruction of exercises to strengthen muscles responsible for moving the shoulders forwards and backwards.

Postoperative exercises and physiotherapy procedures in groups A and B were carried out as follows:

1. Techniques to cleanse the lungs including mobilization, manual techniques, use of the active cycle of breathing techniques and use of IS;
2. Auxiliary active movements of the extremities;
3. Active movement of the extremities;
4. Breathing exercises and expansion of the lobes of the lungs.

In group A during the two-week period before the surgical operation, 15 sessions were held, consisting of exercises and auxiliary activities for extension and rotation of thoracic vertebrae, breathing exercises, exercises to expand lung lobes, instruction of Incentive Spirometer (IS) equipment, extension exercise for thoracic cavity muscles and muscles with a role in breathing (aerobic exercises) for 25 minutes at a constant low speed for all the patients. The patients once again underwent physiotherapy for the respiratory system. However, patients in group B, received physiotherapy only after surgery.

### Outcome measurements

Evaluation of respiratory function was carried out by trained physiotherapists, using spirometry parameters and arterial blood gases (ABGs).

In group A, spirometry was carried out 15 days before surgery and immediately after discharge from the ICU. It should be notified that ICU routine set up was performed for every patient and ventilator was set as Synchronized Intermittent Mandatory Ventilation (SIMV). In group B, spirometry was also carried out before surgery and immediately after discharge from the ICU, and the parameters were compared. In addition, during the stay in the ICU, ABG parameters were compared between the two groups.

Spirometry parameters included forced expiratory volume in 1 s (predicted FEV1), predicted forced vital capacity (FVC), predicted peak flow and peak expiratory flow (PEF). Spirometry was carried out for group A (intervention group) once before rehabilitation programs and once after discharge from the ICU. For group B however, it was performed once 15 days before CABG and once immediately after discharge from the ICU.

ABG parameters consisted of partial pressure of carbon dioxide (PCO_2_), partial oxygen pressure (PO_2_), bicarbonate (HCO_3_) and oxygen saturation (O_2_ sat). ABG parameters were measured once at entry into ICU and once at discharge from the ICU for both groups.

### Data analysis

In a pilot study on 10 patients, the reliability of the observer as well as the reliability of the equipment were evaluated, separately. Kappa coefficient was used to estimate the reliability of the study, which was calculated to be more than 90% in both evaluations.

Student’s t-test analyses were used to compare the study variables at a confidence interval of 95%, using SPSS.

## Results

### Flow of participants through the trial

Sixty seven patients as candidates for open cardiac surgery using the mid-sternotomy technique were screened for eligibility between October 2011 and November 2012. In the present clinical trial, 60 patients were randomly assigned to groups A (n=30) and B (n=30). Seven patients were excluded from the study for various reasons including arthritis, dissatisfaction and lack of access (Figure 1). The groups A and B had similar baseline demographic characteristics and difference between them was not statistically significant [age: P=0.096; sex: P=0.97; smoking: P=0.51; diabetes mellitus: P=0.51; body mass index (BMI): P=0.29, duration of operation: P=0.82; Table 1].

**Table 1:** Baseline variables of patients who completed the study

**Variables**	**Groups**		**P-Value**
	**Expression (n=30)**	**Control (n=30)**	
Age; Years, mean (SD)	54.4 (10.8)	59.3 (10.45)	0.096
Gender; Female, frequency (%)	11 (36.7)	10 (33.3)	0.97
Smoking; Yes, frequency (%)	9 (30)	10 (33.3)	0.51
Diabetes mellitus; Yes, frequency (%)	11 (36.7)	8 (26.6)	0.29
Body Mass index (BMI); mean (SD)	26.8 (4.7)	27.7 (4.9)	0.46
Duration of operation, Hours ; mean (SD)	2.6 (0.23)	2.8 (.36)	0.82

### Effect of intervention

After data normality was confirmed, making it possible to use parametric tests, Student’s t-test was used to compare the means of hours of stay in the ICU in group A (45.9±17.9 h) and group B (69.9±26.3 h) patients, which revealed significant differences between the two groups (mean difference: -24.0, 95% CI: -36.19 to -12.87). Duration of mechanical ventilation (MV) use was defined from the time the patient entered the ICU to the moment of extubation. With the following results, the means of MV for patients in groups A and B were 10.6±3.8 and 17.2±4.9 h, respectively, with significant differences between the two groups (mean difference: -6.6 h, 95% CI: -8.84 to -4.22). In order to neutralize the effect of each patient’s initial status in relation to respiratory function, spirometry and ABG parameters were measured in both groups before rehabilitation and surgery. The two groups were evaluated and compared regarding the differences in each parameter before and after rehabilitation per patient (Table 2). Evaluation of spirometry parameters by mean difference analysis showed significant changes in predicated FVC (CI 95%: 1.3 to 8.7) and PEF parameters (CI 95%: 1.98 to 9.4) in the group A compared to the group B (Table 2). Mean difference of PCO_2_ concentration (of ABG parameters) in group A was significantly more than that in group B (CI 95%: 1.43 to 8.9). It was observed no difference in the other parameters of ABG (pH; CI 95%: -23.9 to 21.3, PO_2_; CI 95%: -23.9 to 21.3, HCO_3_; CI 95%:-3.1 to .26 and O_2_ sat; CI 95%: -3.2to 12.7) between two groups.

**Table 2:** Mean (SD) for all outcomes for each group, mean (SD) difference within groups, and comparison (95% CI) between groups

**Outcome**		**Groups**				** Difference within groups**		**Difference between interventions**
		**Preoperative**		**Post operative**		**Post minus pre-operative**		**Post minus pre-operative**
		**Expression** **(n=30)**	**Control** **(n=30)**	**Expression** **(n=30)**	**Control** **(n=30)**	**Expression** **(n=30)**	**Control** **(n=30)**	**Expression- Control**
**Spirometry indicators**	FEV1	81.7 (13.1)	79.1 (13.4)	80.03 (12.4)	73.8 (13.05)	-1.4 (2.2)	-5.3 (10.9)	3.9 (-0.46 to 7.9)
	FVC	85.8 (9.7)	81.1 (10.6)	84.5 (8.96)	74.7 (12.8)	-1.37 (2.4)	-6.4 (9.8)	5.03* (1.3 to 8.7)
	PEF	68.3 (16)	74.0 (15.9)	68.5 (14.3)	68.7 (16.5)	0.2 (7.2)	-5.5 (7.2)	5.7 * (1.979 to 9.4)
ABG parameters	pH	7.3 (0.08)	7.3 (0.06)	7.38 (0.05)	7.39 (0.034)	0.08 (0.086)	0.09 (0.07)	-0.01 (-0.05 to 0.031)
	PCO_2_	37.1 (6.3)	39.8 (7.7)	41.57 (3.44)	39.1 (3.9)	4.47 (5.99)	-0.7 (8.31)	5.17* (1.43 to 8.9)
	PO_2_	128 (54.1)	106.9 (24.5)	149.9 (34.9)	126.27 (40.73)	21.9 (42.76)	19.37 (44.7)	2.53 (-23.9 to 21.3)
	HCO_3_	21.7 (2.6)	20.2 (2)	22.6 (1.668)	22.83 (1.82)	0.9 (5.01)	2.63 (2.156)	-1.73 ( -3.1 to 0.26)
	O_2_ Sat	95.0 (3.5)	97.0 (2.8)	105.4 (13.49)	102.6 (18.66)	10.4 (12.99)	5.6 (17.3)	4.8 (-3.2 to 12.7)

ABG: Arterial blood gas

## Discussion

Although some studies have emphasized the effect of rehabilitation on the efficacy of the respiratory system after surgery, only limited number of studies have evaluated the effect of respiratory rehabilitation before surgery.^8-12^

In this randomized clinical trial, the duration of stay in the ICU, duration of use of MV, spirometry and ABG parameters were used in order to evaluate the effect of preoperative physiotherapy on the respiratory performance.

One of the benefits of preoperative respiratory physiotherapy compared to postoperative one was its positive effect on the duration of patient hospitalization that was inconsistent with the results of some studies.^12-15^

Another beneficial effect of the intervention in this study was a decrease in the duration of MV using and inpatient in ICU and then decrease in the costs inflicted on the patients. The intervention also minimized the possibility of MV complications and accelerated the speed of patient recovery. These results agreed with some studies in which preoperative physiotherapy before both open-heart surgery has been found to reduce the hospital stay.^12,16^

Of the spirometry parameters in the present study, differences were observed in the means of FVC and PEF between intervention and control groups; however, as expected, the difference in the means was higher in group A compared to the group B, which is consistent with the results of some other studies.^14,17^

Evaluation of respiratory performance based on ABG parameters showed significantly greater differences in the means of PCO_2_ in the intervention group. Preoperative physiotherapy before open-heart surgery has been found to diminish blood gases and to improve the quality of life.^12^

Due to a lack of sufficient studies on the subject, the conduction of more studies deems necessary in relation to the effect of preoperative rehabilitation on the respiratory function based on ABG parameters. The small sample size, loss of a number of cases due to the distance between home and treatment center and impossibility of accommodation of patients and Lack of measuring quality outcomes were of limitations of the study.

## Conclusion

The results of this study based on the evaluation of parameters of respiratory performance showed that preoperative respiratory physiotherapy could have a positive effect on the improvement of quality of respiratory performance in patients undergoing open cardiac surgery. Further evaluations are necessary in relation to the sensitivity and specificity of spirometry parameters along with the evaluation of respiratory performance.

## Ethical issues

The study protocol was approved by the local Ethics Committee under the code of 2272/4/5 and registered in the International Center for Clinical Trials under the code number IRCT138904294422N1 (http://www.irct.ir). All participants gave written informed consent before data collection began.

## Competing interests

Authors declare no conflict of interests in this study.
